# Revealing New Mouse Epicardial Cell Markers through Transcriptomics

**DOI:** 10.1371/journal.pone.0011429

**Published:** 2010-06-28

**Authors:** Lars Bochmann, Padmini Sarathchandra, Federica Mori, Enrique Lara-Pezzi, Domenico Lazzaro, Nadia Rosenthal

**Affiliations:** 1 Mouse Biology Unit, European Molecular Biology Laboratory, Monterotondo, Italy; 2 Harefield Heart Science Centre, Imperial College London, London, United Kingdom; 3 Istituto di Ricerche di Biologia Molecolare P. Angeletti SpA, Pomezia, Italy; 4 Cardiovascular Development Biology Department, Centro Nacional de Investigaciones Cardiovasculares – CNIC, Madrid, Spain; Istituto Dermopatico dell'Immacolata, Italy

## Abstract

**Background:**

The epicardium has key functions during myocardial development, by contributing to the formation of coronary endothelial and smooth muscle cells, cardiac fibroblasts, and potentially cardiomyocytes. The epicardium plays a morphogenetic role by emitting signals to promote and maintain cardiomyocyte proliferation. In a regenerative context, the adult epicardium might comprise a progenitor cell population that can be induced to contribute to cardiac repair. Although some genes involved in epicardial function have been identified, a detailed molecular profile of epicardial gene expression has not been available.

**Methodology:**

Using laser capture microscopy, we isolated the epicardial layer from the adult murine heart before or after cardiac infarction in wildtype mice and mice expressing a transgenic IGF-1 propeptide (mIGF-1) that enhances cardiac repair, and analyzed the transcription profile using DNA microarrays.

**Principal Findings:**

Expression of epithelial genes such as basonuclin, dermokine, and glycoprotein M6A are highly enriched in the epicardial layer, which maintains expression of selected embryonic genes involved in epicardial development in mIGF-1 transgenic hearts. After myocardial infarct, a subset of differentially expressed genes are down-regulated in the epicardium representing an epicardium-specific signature that responds to injury.

**Conclusion:**

This study presents the description of the murine epicardial transcriptome obtained from snap frozen tissues, providing essential information for further analysis of this important cardiac cell layer.

## Introduction

The epicardium is a single epithelial cell layer overlying the vertebrate heart. It derives from the proepicardium that gives rise to the vasculature and interstitial cells of the heart during embryogenesis, a process that is mandatory for normal development [1], [2], [3], [4]. It is still under debate if the epicardium also gives rise to endothelial cells of the vasculature [5], [6] and if it contributes to the myocardium directly [1], [2], [7]. Deletions of selected genes expressed in the epicardium (i.e. VCAM-1, α_4_-integrin) or signalling from the myocardium to the epicardium (i.e. Tβ4, FOG-2) lead to severe defects in the developing heart and its vasculature [3], [6].

Beyond its role during cardiac development, the epicardium also mediates cardiac regeneration after injury in lower vertebrates. Specifically, the zebrafish epicardium supports cardiac regeneration through epithelial to mesenchymal transition (EMT) and subsequent migration into the myocardium to form new vasculature [8]. In mammals, these regenerative processes are not active to the degree that they support recovery of infarcted myocardial tissue. Instead, myocardial infarction is followed by inflammation and scarring. The fibrotic tissue is not able to support normal cardiac function, which in many cases leads to cardiac failure. However, the epicardium does retain some capability to be involved in repair processes also in mammals, in that for example Thymosin beta-4 can activate adult epicardial cells [9] acting through reactivation of embryonic signalling pathways [10]. This thymosin mediated activation of epicardial cells can in fact support revascularization of the injured mammalian heart by forming endothelial and vascular smooth muscle cells [11]. Further, a sub-population of adult epicardial cells retains the potential to give rise to cardiac precursors or endothelial cells [12]. The regenerative potential of the epicardium has also prompted several other strategies to improve mammalian cardiac regeneration, including the injection of epicardium-derived cells (EPDCs) into the injured myocardium, which was reported to enhance cardiac repair, highlighting their potential clinical importance [13]. In newer studies cardiomyocyte progenitors were cotransplanted with EPDCs into infarcted myocardial tissues, which improved functional repair when compared to single cell type supplementation [14]. The effect however was shown to be caused by paracrine effects from both cell types.

Although some of the genes involved in epicardial function have been identified and characterised [15], [16], [17], comprehensive transcriptional analyses of the adult mammalian epicardium have been lacking. In this study, we employed laser capture microscopy (LCM) on snap frozen tissues to obtain unaltered epicardial sample. This micro-dissection technique afforded a very precise excision of the epicardial layer without prior modification, such as labelling of epicardial cells, which may compromise RNA quality; without cell sorting, which might isolate only a particular cell fraction; and without cell culturing, thereby avoiding inevitable changes in gene expression.

Using this approach, we identified numerous genes that have not previously been implicated in the function, homeostasis, or regeneration of the epicardium or the heart. Our results provide several markers of the steady state adult epicardium, and changes of epicardial signature gene expression in response to LCA injury that underscore the involvement of the epicardium after cardiac damage. Epicardial signature gene expression patterns were altered in a model of enhanced cardiac regeneration by delivery of a localized transgenic IGF-1 propeptide, which reactivated genes implicated in the effective regenerative response of other vertebrate species. This study provides a wealth of information on gene expression patterns in this important tissue, which will prove potentially valuable in future studies of epicardial function in cardiac homeostasis and infarction models.

## Results

### Transcripts encoding several genes are enriched in the epicardium

**Figure 1:**
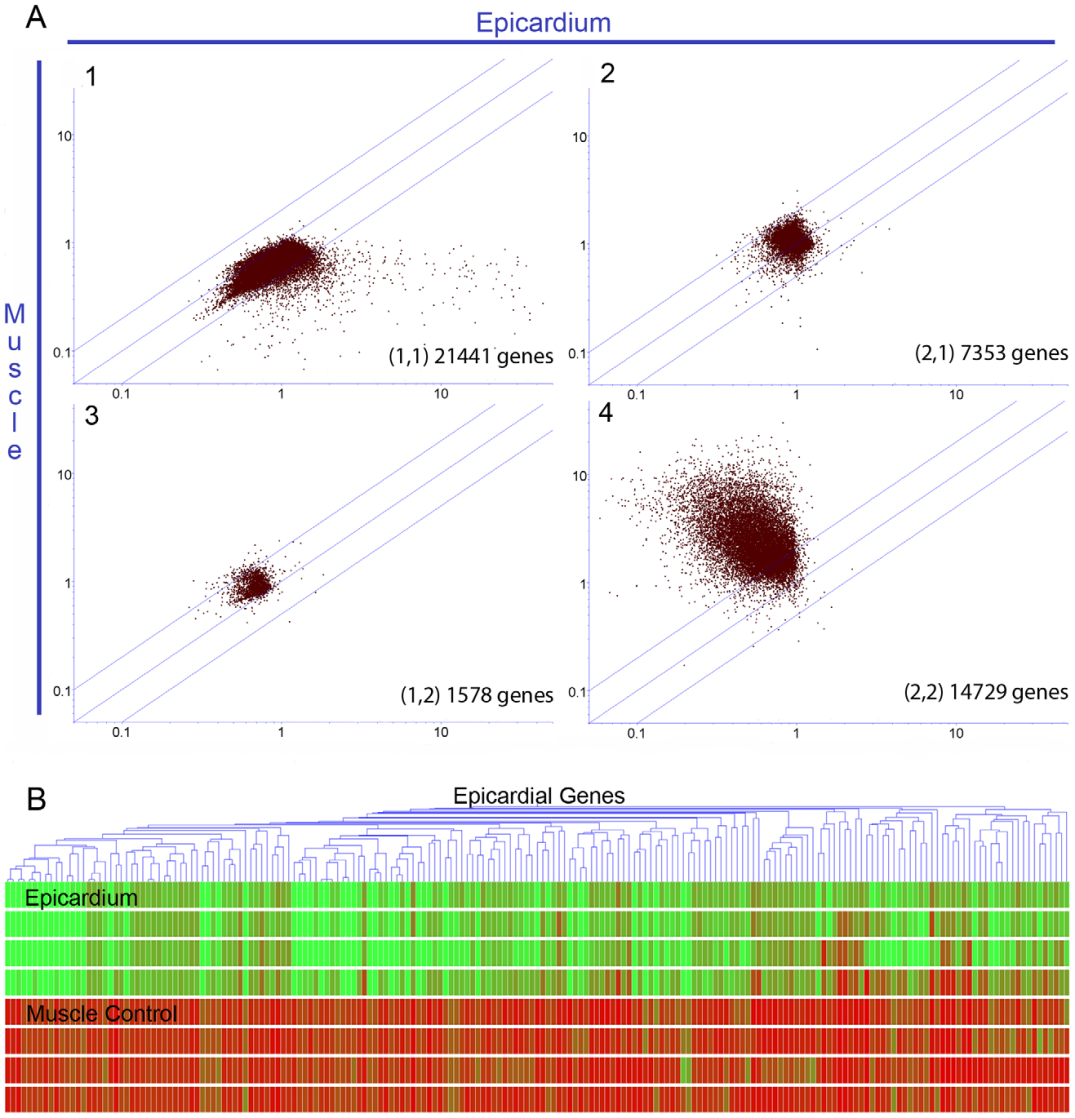
Unsupervised analysis of epicardial gene expression. A self organizing map was used as an unsupervised analysis tool to simplify complex expression information. B) x-axis are relative normalized expression of epicardium samples, y-axis are relative normalized expression of muscle samples. The four resulting clusters represent generally different expression patters rather than their absolute expression values. Cluster 1 contains genes that were higher in the epicardial samples than in the muscle controls. Clusters 2 and 3 contains genes that were similar in expression in epicardium and muscle. Cluster 4 contains genes that were higher in the muscle controls than in the epicardial samples. B) Starting from Cluster 1, we applied conditions as described in Materials and Methods to select 197 epicardial signature genes shown as a heat map. The top four rows are epicardial samples, and the lower four rows are muscle control samples, where green colour represents upregulation and red colour represents downregulation of a given gene.

Using LCM technology, we isolated the left ventricular epicardial layer of frozen murine hearts as described in Materials and Methods (Figure S1), resulting in an epicardium-enriched sample. RNA isolated from epicardial and underlying myocardial tissue samples were compared by DNA microarray analysis (Array Express: E-MEXP-2446). To gain an overview of differential gene expression between the epicardial and muscle control samples, we performed an unsupervised analysis of the gene array data (SOM 2×2). This resulted in four clusters (Figure 1-A). The first cluster was populated by genes that were higher in epicardial samples than in muscle samples, the second and third cluster were mainly unchanged among the samples, and the fourth cluster contained genes that were higher in the non-epicardial muscle control samples. To establish criteria for epicardial genes we applied conditions based on Cluster 1, to define genes that are highly expressed in the epicardium but not in the muscle controls thus representing the epicardial signature genes, as described in Materials and Methods (Table 1, Table S1). These genes are also shown as a heat map (Figure 1-B).

**Table 1:** Epicardial Genes.

Fold difference	Gene Bank accesion	Gene Symbol	Gene Description
269.6	BQ084786	Upk3b	uroplakin 3B
199.4	BB348674	Gpm6a	glycoprotein m6a
129.8	BI452905	Dmkn	dermokine
88.97	AV290571	C2	complement component 2 (within H-2S)
81.48	BB427704	Upk1b	uroplakin 1B
76.86	NM_026228	Slc39a8	solute carrier family 39 (metal ion transporter), member 8
68.68	AK004699	Cyp2s1	cytochrome P450, family 2, subfamily s, polypeptide 1
56.12	BB118542	Slc26a3	solute carrier family 26, member 3
53.11	BB325766	Lrrn4	leucine rich repeat neuronal 4
45.18	NM_031170	Krt8	keratin 8
45.14	NM_011315	Saa3	serum amyloid A 3
42.83	NM_008471	Krt19	keratin 19
37.65	AW556821	2610018G03Rik	RIKEN cDNA 2610018G03 gene
36.93	NM_013473	Anxa8	annexin A8
35.12	K02782	C3	complement component 3
33.93	AJ132433	Prr15	proline rich 15
33.63	BB805362	Slc9a3r1	solute carrier family 9 (sodium/hydrogen exchanger), member 3 regulator 1
31.76	BC005611	Chi3l1	chitinase 3-like 1
31.32	BB392676	1500015O10Rik	RIKEN cDNA 1500015O10 gene
30.71	U88064	Bnc1	basonuclin 1
29.94	AK003577	Muc16	mucin 16
28.49	NM_008344	Igfbp6	insulin-like growth factor binding protein 6
28.24	BC023060	Efemp1	epidermal growth factor-containing fibulin-like extracellular matrix protein 1
26.95	NM_021426	Nkain4	Na+/K+ transporting ATPase interacting 4
26.57	BC015076	Mpzl2	myelin protein zero-like 2
26.08	AI852300	Ildr2	immunoglobulin-like domain containing receptor 2
…	…	…	…
8.79	NM_00902	Aldh1a2	Aldehyde dehydrogenase family 1
5.15	AK012980	Tbx18	T-box18

List of epicardial genes expressed in epicardial samples isolated by LCM as described in Materials and Methods. Genes from Cluster 1 (from Figure 1), were selected for statistically significant upregulation in the epicardial samples, a normalized >4-fold increased expression in the epicardial sample compared to the muscle control and raw expression of at least 20 in the epicardial samples. This selection identified 197 unique epicardial signature genes. This Table includes top hits with a cut off at 25 fold induction in epicardial samples plus two selected epicardial genes with lower induction.

As a separate quality control, we also compared the epicardial sample to left ventricle or whole heart tissues [18], by comparing existing gene array data and applying the criteria for epicardial genes (starting from all genes, t-test, multiple testing correction, 4 fold upregulation, raw in epicardium above 20) to the pairs: epicardium/heart, epicardium/left ventricle, and epicardium/muscle control. We then compared the resulting lists of epicardial signature genes. Although the lists were not identical, especially for the highly epicardium specific genes, the results were consistent, in that the genes from our original epicardial signature genes in Table 1 (epicardial with >25 fold induction in the epicardium) were all found in all three lists of epicardial genes (Figure S2, Table S2). Differences were mainly observed in genes with lower induction.

**Figure 2:**
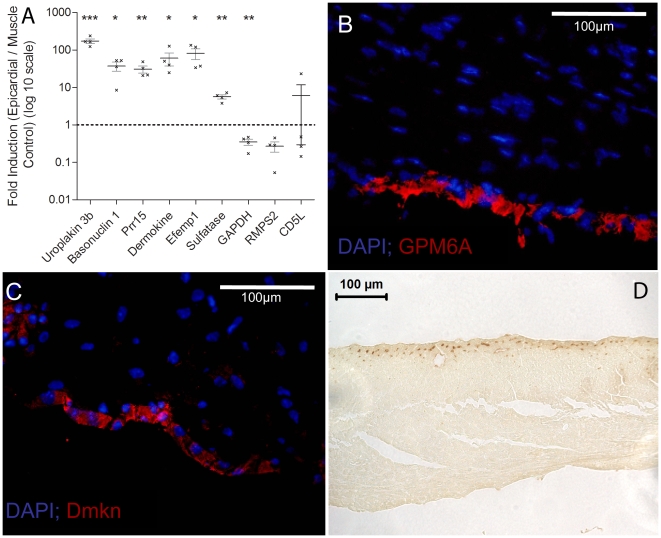
Confirmation of epicardial gene expression. A) qRT-PCR was used to confirm gene array data, normalized against 18 S expression. Statistical significance was tested using the student t-test. Expression levels for uroplakin 3b, basonuclin 1, proline rich 15, dermokine, Efemp1, and sulfatase were significantly upregulated in the epicardial samples. GAPDH, RMPS2, and CD5L serve as controls; GAPDH was expressed higher in muscle tissue while RMPS2 and CD5L showed no statistically relevant expression in the different samples as expected. B,C) Using immunohistochemical localization of glycoprotein M6A (B), dermokine (C), and basonuclin-1 (D) proteins in the epicardial or subepicardial layer. (* denotes statistical significance with p<0.05, **: p<0.005, ***: p<0.0005; Error bars are S.E.M., scale bars are 100 microns).

This analysis uncovered numerous epicardial signature genes such as uroplakin 1b, 3b, glycoprotein m6a, dermokine or basonuclin that have not previously been implicated in the function, homeostasis or regeneration of the epicardium or the heart. Importantly, our list of epicardial signature genes also contained several genes whose expression have previously documented in the epicardium, playing key roles in epicardial development. Among these were RALDH2 (9 fold increase) the earliest retinoic synthetic enzyme in the embryo and implicated in mammalian epicardial contribution to the coronary vasculature and myocardial repair in the zebrafish system [8], [19], [20], [21]. The previously documented expression of Tbx18 in epicardial cells as well as epicardial progenitors [2], also validates the epicardial identity of our signature gene collection. Selective quantitative RT-PCR analysis for dermokine, basonuclin-1, Efemp-1, proline rich 15, sulfatase 1, and uroplakin-3b transcripts, confirmed epicardial enrichment (Figure 2-A). To confirm these observations on the protein level, immunohistochemical analysis localized dermokine, basonuclin-1, and GPM6A to the epicardium (Figure 2-B,C).

Systematic analysis of tissue expression profiles by comparing the list of epicardium-enriched transcripts to relevant gene ontology lists (GO: 8150; biological process, see Materials and Methods), revealed mainly immune system-related lists (i.e. GO: 2526, 2541, 6956, 6692, 45087, 6959) with very low p-values. Notably, the list for extracellular matrix organization (GO: 30198) as well as the cell adhesion and biological adhesion lists (GO: 7155, 22610) also had a high similarity (p<0.0005) to the epicardial gene list from normal (un-injured) hearts (Table 2, Table S3).

**Table 2:** GO ontology analysis of epicardial signature genes.

GOID	Term	q	m	p
GO:0002526	acute inflammatory response	9	141	5.44*10∧–7
GO:0002541	activation of plasma proteins involved in acute inflammatory response	6	70	1.36*10∧–5
GO:0006956	complement activation	6	70	1.36*10∧–5
GO:0006692	prostanoid metabolic process	4	23	2.15*10∧–5
GO:0006693	prostaglandin metabolic process	4	23	2.15*10∧–5
GO:0045087	innate immune response	8	168	2.72*10∧–5
GO:0048869	cellular developmental process	36	3337	5.51*10∧–5
GO:0006959	humoral immune response	6	90	5.73*10∧–5
GO:0051605	protein processing by peptide bond cleavage	7	138	6.89*10∧–5
GO:0006958	complement activation, classical pathway	5	59	8.15*10∧–5
GO:0002455	humoral immune response mediated by circulating immunoglobulin	5	64	0.000123
GO:0007155	cell adhesion	18	1092	0.000123
GO:0022610	biological adhesion	18	1094	0.000123
GO:0002253	activation of immune response	7	154	0.00013
GO:0006954	inflammatory response	10	381	0.000287
GO:0030154	cell differentiation	33	3208	0.00035
GO:0002684	positive regulation of immune system process	10	393	0.000371
GO:0016485	protein processing	7	183	0.000395
GO:0048856	anatomical structure development	40	4332	0.000411
GO:0030198	extracellular matrix organization	7	188	0.000465
GO:0032502	developmental process	47	5527	0.00048

Epicardial GO ontology list included various innate immune system lists as well as the cell adhesion list, confirming the epithelial character of the epicardium. An FDR cut off of 0.0005 was used. (q: number of epicardial genes present in the given GO list; m: total number of genes in a given GO list; p: FDR value for a given GO list enrichment among the epicardial genes).

### Epicardial genes are differentially regulated after myocardial infarction

**Figure 3:**
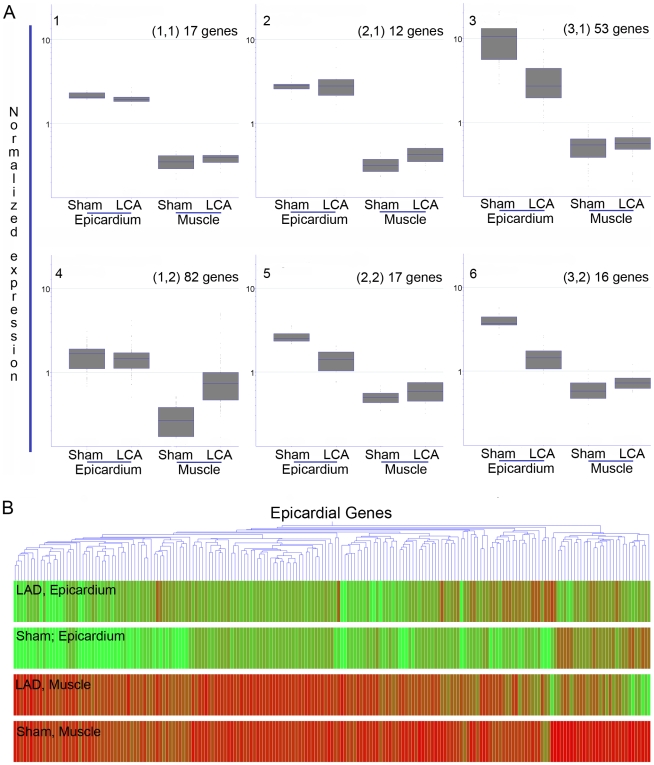
Unsupervised analysis of epicardial gene expression post infarction. A) Self- organizing maps were used to derive six clusters two of which, (3 and 4) contain genes that are strongly regulated. Cluster 3, includes epicardial signature genes whose expression decreases sharply in the epicardium but remains low in muscle post-infarction. Cluster 4 contains epicardial signature genes that are largely unchanged in the epicardium but are increased in muscle post-infarction. B) Heat map of data in A).

To assess the early changes in epicardial gene expression in response to injury, we collected hearts from a mouse model of myocardial infarction induced by left coronary artery ligation (LCA) and compared to those of non-infarcted (sham operated) mice. Epicardial samples and muscle controls for both conditions were obtained using LCM as described above, and changes in expression levels were analyzed six days after myocardial infarction using gene arrays. SOM cluster analysis as described in Materials and Methods for the previously determined epicardial genes (Figure 3-A), are summarized in a heat map (Figure 3-B). Among the six obtained clusters, Cluster 3 contained 53 epicardial signature genes that decreased steeply after infarction, with only slight changes in the underlying cardiac muscle control samples. Conversely, Cluster 4 contained 82 signature genes whose expression was relatively stable for the epicardial samples, while their expression in underlying muscle increased significantly after infarction. The remaining clusters contained mainly slight variations of these two patterns, or no significant changes. We carried out statistical analysis for Clusters 3 and 4, resulting in a list of 23 signature genes from Cluster 3 that were significantly downregulated in the epicardial samples (Table 3) and a list of 21 signature genes from Cluster 4 that were significantly upregulated in the muscle samples post-infarction (Table 4). When carrying out GO ontology analysis on the list of Cluster 3 signature genes downregulated in the infarcted epicardium, we observed similar enrichment of GO lists as for the initial epicardial gene analysis, such as acute inflammatory response (GO:2526), complement activation (GO:6956), innate immune response (45087), and inflammatory response (GO:2541) (Table S4). These lists were consistently populated by complement-related genes and many of the signature genes enriched in the uninjured epicardium (11 downregulated genes were among those 26 signature genes with a fold difference above 25, while the other 12 were among the remaining 171 signature genes with a lower fold induction).

**Table 3:** Downregulated epicardial signature genes after myocardial infarction.

p-value	GenBank accession	Gene Symbol	Gene Description
0.0474	U88064	Bnc1	basonuclin 1
0.045	NM_021719	Cldn15	claudin 15
0.0356	AV347903	Gng4	guanine nucleotide binding protein (G protein), gamma 4
0.0286	AK004699	Cyp2s1	cytochrome P450, family 2, subfamily s, polypeptide 1
0.0241	BE945607	Cybrd1	cytochrome b reductase 1
0.0241	NM_008198	Cfb	complement factor B
0.0208	AV288135	Atad4	ATPase family, AAA domain containing 4
0.0208	BB348674	Gpm6a	glycoprotein m6a
0.0208	NM_026228	Slc39a8	solute carrier family 39 (metal ion transporter), member 8
0.0208	BB325766	Lrrn4	leucine rich repeat neuronal 4
0.0208	AK003577	Muc16	mucin 16
0.0208	NM_021426	Nkain4	Na+/K+ transporting ATPase interacting 4
0.0208	BC023060	Efemp1	epidermal growth factor-containing fibulin-like extracellular matrix protein 1
0.0142	K02782	C3	complement component 3
0.00681	BC022950	1600029D21Rik	RIKEN cDNA 1600029D21 gene
0.00681	BC010782	Tm4sf5	transmembrane 4 superfamily member 5
0.00616	NM_008365	Il18r1	interleukin 18 receptor 1
0.00616	NM_138683	Rspo1	R-spondin homolog (Xenopus laevis)
0.00616	BB075402	Zdbf2	zinc finger, DBF-type containing 2
0.00249	AV012073	Rarres2	retinoic acid receptor responder (tazarotene induced) 2
0.00217	BB118542	Slc26a3	solute carrier family 26, member 3
0.00215	NM_007753	Cpa3	carboxypeptidase A3, mast cell
0.00196	AV144145	Chrdl1	chordin-like 1

Epicardial signature genes of Cluster 3 (Figure 3) were tested for statistically significant downregulation in the epciardium samples as described in Materials and Methods. This list shows those genes that were downregulated (p value cut off: 0.005) in the epicardium samples.

**Table 4:** Epicardial signature gene expression changes in muscle after myocardial infarction.

p-value	GenBank accession	Gene Symbol	Gene Description
0.0497	BB558905	Bace2	beta-site APP-cleaving enzyme 2
0.0495	NM_033314	Slco2a1	solute carrier organic anion transporter family, member 2a1
0.0473	NM_009242	Sparc	secreted acidic cysteine rich glycoprotein
0.0433	BF235516	Ptprf	protein tyrosine phosphatase, receptor type, F
0.0433	AK011545	Basp1///LOC100045716	brain abundant, membrane attached signal protein 1///similar to 22 kDa neuronal tissue-enriched acidic protein
0.0359	BB065799	Sulf1	sulfatase 1
0.0329	U08020	Col1a1	collagen, type I, alpha 1
0.0329	L36062	Star	steroidogenic acute regulatory protein
0.0329	NM_010329	Pdpn	podoplanin
0.0259	BF227507	Col1a2	collagen, type I, alpha 2
0.0211	AW550625	Col3a1	collagen, type III, alpha 1
0.0211	NM_011254	Rbp1	retinol binding protein 1, cellular
0.0211	NM_009636	Aebp1	AE binding protein 1
0.0175	BB392676	1500015O10Rik	RIKEN cDNA 1500015O10 gene
0.0175	NM_011985	Mmp23	matrix metallopeptidase 23
0.0175	NM_013586	Loxl3	lysyl oxidase-like 3
0.0175	X16834	Lgals3	lectin, galactose binding, soluble 3
0.0174	BC022666	Mfap4	microfibrillar-associated protein 4
0.0147	BC008107	Timp1	tissue inhibitor of metalloproteinase 1
0.00893	M65143	Lox	lysyl oxidase
0.00742	BC025600	Tmem119	transmembrane protein 119

Epicardial signature genes of Cluster 4 (from Figure 3) were tested for statistically significant upregulation in the muscle control samples as described in Materials and Methods. This Table shows those genes that were upregulated (p value cut off: 0.005) in the muscle control samples.

Interestingly, GO ontology analysis for epicardial signature genes upregulated in the muscle control revealed a distinctly different set of lists (Table S5) resembling mainly developmental and vascularisation processes. Among these, some of the highest ranking lists were collagen fibril organization (GO:301099), extracellular matrix organization (GO:30198), skin morphogenesis (GO:43589), vasculature development (GO:1944), epidermis morphogenesis (48730), or blood vessel development (GO:1568). Among the highly specific epicardium signature genes, which were downregulated post-infarct, only one of the epicardial signature genes was increased in the muscle post-infarct (Riken 1500015O10, induction of 31 fold).

We also examined a selected subset of epicardial genes most significantly affected by infarction for further analysis at an earlier time point (three days post-infarction). RT-PCR analysis of three and six day post-infarction epicardial samples showed a consistent downregulation of uroplakin 3b, proline rich 15, dermokine, Efemp-1, sulfatase-1, and basonuclin compared to sham operated hearts (Figure S3). Conversely, CD5L was strongly upregulated 3 days post infarction but subsided at six days, highlighting the importance of immune system related genes in early infarct response processes, and confirming the specificity of changes are in the post-infarct epicardium.

Regarding less dramatically affected genes than the epicardial signature subset, we observed a trend towards up-regulation of vimentin, fibronectin, snail2, and MMP-2 as well as a modest down-regulation of desmoplakin in the epicardial transcriptome after injury (data not shown), all of which are associated with cells undergoing epithelial-to-mesenchymal transition [22]. Thymosin β-4, a protein implicated in the activation and mobilization of adult epicardial cells [9], and Thymosin β-10, a potent inhibitor of angiogenesis [23], were both slightly increased (data not shown). Similar changes were observed in the underlying cardiac muscle samples, indicating a general response of these genes to injury.

### Epicardial expression changes in mIGF-1 enhanced cardiac regeneration

We have previously shown that supplemental transgenic expression of a locally acting Insulin-like Growth Factor-1 propeptide (mIGF-1) in cardiomyocytes improved repair of the heart after infarction. Restoration of cardiac form and function in post-infarct mIGF-1 transgenic mice was facilitated by modulation of the inflammatory response [30]. To determine how cardiac mIGF-1 expression changes the transcription profile of the pre- and post- infarct epicardium, we compared the expression of mIGF-1 transgenic to wildtype epicardial samples in normal and infarcted hearts. In analyses of the mIGF-1 signature gene list (Table S6) by GO ontology, genes that were found only in the mIGF-1 epicardium were represented in RNA processing GO lists such as mRNA processing (GO: 6397) or mRNA metabolic processes (GO: 16071), in contrast to genes only in the wildtype epicardium represented in acute inflammatory response (GO:2526), complement activation (GO: 2541), or innate immune response (GO: 45087).

Although the most highly expressed genes of the unperturbed epicardium were common to both genotypes, several genes were selectively upregulated in the epicardium of mIGF-1 animals. Comparing wildtype and mIGF-1 samples revealed a number of genes that were highly epicardium specific in the mIGF-1 samples but that were not part of the epicardial signature in the wildtype mice (Table 5). Furthermore, two known epicardial genes were increased in the epicardium of mIGF-1 transgenic mice when compared to wildtype samples, namely Tbx18 (8.5 fold increase) and RALDH2 (40 fold increase). Thus, the epicardium of mIGF-1 transgenic mice differs in its normal gene expression profile from the wildtype hearts, suggesting a role for the epicardium in the regenerative effects of mIGF-1 expression from the underlying myocardium.

**Table 5:** New epicardial genes induced in mIGF-1 hearts.

Fold Difference	Gene Symbol	Gene Description
43.96	Pik3cd	phosphatidylinositol 3-kinase catalytic delta polypeptide
34.41	Ptprk	protein tyrosine phosphatase, receptor type, K
30.04	Rbbp8	retinoblastoma binding protein 8
27.13	Polr3k	Polymerase (RNA) III (DNA directed) polypeptide K (Polr3k), mRNA
26.19	Nrip2	nuclear receptor interacting protein 2
25.8	Cryl1	crystallin, lambda 1
25.67	1700003E16Rik	RIKEN cDNA 1700003E16 gene
22.52	H2-T10	histocompatibility 2, T region locus 10
22.26	1110069O07Rik	RIKEN cDNA 1110069O07 gene
20.27	EG620313///Tbca	predicted gene, EG620313///tubulin cofactor A
20.05	Marcksl1	MARCKS-like 1

IGF-1 and WT epicardial samples were compared to the underlying myocardium to examine variations in epicardial signature genes (as defined in Materials and Methods). Several genes that had a >20 fold induction in the epicardium of mIGF-1 hearts were not at all represented in epicardial genes in WT hearts.

**Figure 4:**
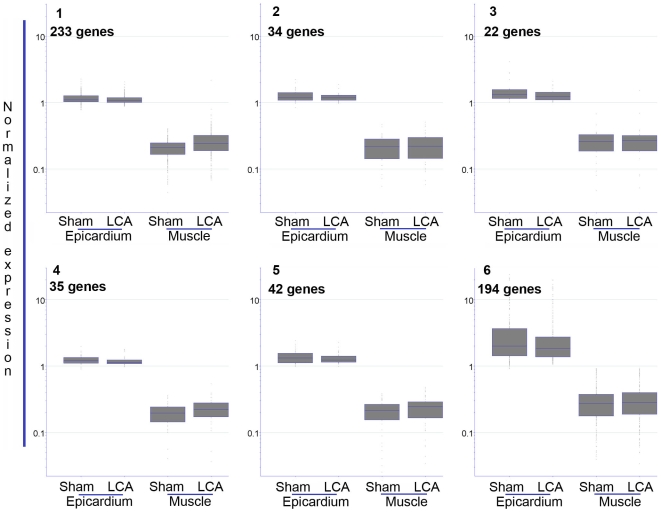
Unsupervised analysis of epicardial gene expression changes post infarction in IGF-1 animals. Self- organizing maps were used to derive six clusters of epicardial genes for IGF-1 overexpressing mice. Unlike for the wildtype animals, no cluster contained extensive expression changes post infarction.

To test this possibility, we compared the expression profiles of the wildtype and mIGF-1 hearts six days post infarction. Interestingly, the post-infarction gene expression changes seen in wildtype animals were less evident in the mIGF-1 hearts: unsupervised analysis of mIGF-1 epicardial genes using SOM revealed no cluster with significant changes of expression (Figure 4), indicating that the action of mIGF-1 alleviates the transcriptional perturbations caused by myocardial infarction.

## Discussion

The relative paucity of cell-restricted markers represents a current constraint on physiological and pathophysiological studies of the adult murine epicardium. A transcriptomic approach to elucidating epicardial marker genes is a powerful way to obtain better insight into the expression profile and characteristics of the epicardial layer. Using laser capture microscopy, we developed an analytical approach for the identification of novel epicardial genes, yielding a list of potentially interesting genes for epicardial function that has not been possible with more traditional, less high-throughput approaches. The use of LCM affords the unbiased collection of the entire cell population of the epicardium/subepicardium as opposed to more selective cell sorting or staining-based technologies. Although we confirmed the microarray results for an epicedial signature gene subset at the protein level, any analysis based on RNA-transcripts has its limitations, and more extensive validation of protein expression will be necessary when examining other genes contained in these lists.

Other approaches have compared gene expression of epicardial regions to endocardial regions of the ventricular wall of the rat [24], in this study we focus on the comparison of gene expression of the murine epicardium to the ventricular wall before or after left coronary artery ligation (LCA).

The epicardial transcriptomic analysis presented here highlighted cell adhesion genes characteristic of epithelia, confirming a characterization of the epicardium as an epithelial cell layer covering the heart. On a broader scale, the GO ontology analysis confirms the epithelial character of the epicardium, as exemplified by numerous candidates in the cell adhesion list. Other high scoring GO lists were mainly related to immune response, populated with complement-related genes that are normally expressed in epithelial cells such as keratinocytes of the skin [25] or intestinal epithelial cell lines [26]. The downregulation of these epicardial related immune system genes after infarction further suggests that the epicardium normally expresses immune system-related genes to form a barrier protecting the heart. Of note, an enrichment of GO ontology lists based on the epicardial genes in the infarcted muscle tissue related to developmental processes and particularly in vascularisation and epithelial formation.

Of the novel epithelial genes identified in this study, dermokine and basonuclin are particularly noteworthy in the context of the adult epicardium. Importantly, these genes have been described as epithelial genes in the literature, but heretofore have not been annotated as such in the GO ontology. Dermokine, a gene of unknown function, has been suggested to have a cytokine function, which supports an epicardial signalling role [27]. A recent report that dermokine is up-regulated in canine muscle after sustained endurance exercise [28] suggests a possible involvement in muscle adaptation. Its differential expression in the epicardium after cardiac injury makes dermokine an interesting target for functional studies in acute cardiac response to injury as well as to physiological or pathological hypertrophy.

The transcription factor basonuclin-1 plays a role in epithelial cell differentiation and proliferation and has been studied in a corneal epithelium damage model [29]. Basonuclin-1 null mice display reduced epithelial cell proliferation with thin epidermis and delayed healing of the corneal epithelium. A likely role for this epithelial stem cell marker in epicardial function is the support of cell proliferation [30], a feature necessary for epicardial development and homeostasis. Although basonuclin-null mice are viable, the knockout leads to a decreased level of e-cadherin protein in corneal epithelium, generally associated with increased rates of cell invasiveness and EMT [31]. Should basonuclin play a similar role in the epicardium, regulation of EMT during repair processes of the heart may be compromised in these mice. We are currently investigating this possibility.

Downregulation of epicardial genes as well as the loss of adhesiveness after induction of cardiac infarct is consistent with cells undergoing EMT in the epicardium before myocardial invasion, migration and differentiation into endothelial or smooth muscle cells to form new vasculature. The upregulation of vascularisation and epithelial genes in the muscle tissue further supports this interpretation. Indeed, several markers for EMT were affected in the epicardial as well as cardiac transcriptome after injury. Together with activation of thymosin β-4 expression in the epicardium samples [9], these data suggest that epicardial cells may undergo global activation post-infarction. Without further lineage tracing studies, the fate of epicardial cells after infarction remains uncertain, as epicardial cells might alter their gene expression profile without actually undergoing EMT.

Despite some similarities to gene programs activated in the initial repair processes after resection of zebrafish hearts, the mammalian epicardium cannot support effective regeneration of cardiac damage. In this regard, the shift in epicardial gene expression patterns in mice expressing supplemental mIGF-1 propeptide in the myocardium may support the improved regenerative capacities documented in these transgenic hearts [32]. Specifically, elevated RALDH2 expression in the epicardium in response to supplemental mIGF-1 from the underlying myocardium points to a novel homeostatic re-activation of embryonic programs, in which the pivotal role of retinoic acid in epicardial development is redeployed to prime the tissue for more effective mobilization upon injury. A related study has been published reporting epicardial transcriptional profiling of material obtained by tissue digestion and subsequent physical removal of the epicardial cell layer, confirming post-infarction reactivation of RALDH2 and other embryonic genes that correlate well with the present study [33].These observations will inform further investigation of the altered gene programs elicited by supplemental mIGF-1 propeptides to unravel their role in the enhancement of mammalian cardiac repair. The epicardial signatures presented here provide important tools for such analyses, as well as important information for comparative studies to determine which of the epicardial genes play crucial roles in the development, homeostasis, or repair processes of the heart in other more regenerative organisms.

## Materials and Methods

### Ethics Statement

All mouse procedures were approved by the EMBL Monterotondo Ethical Committee (Monterotondo, Italy) and were in accordance with national and European regulations.

### Animal keeping and Myocardial Infarction model

WT male C57/Bl6 mice were purchased from Charles River Laboratories. Male mIGF-1 transgenic animals were used as described before [32]. All animals used were mus musculus of C57/Bl6 background. Animals were kept in IVC mouse racks at a 12 h/12 h light-dark cycle and were fed with pellet food and drinking water. Bedding material was changed once a week.

Male animals between three and four months of age were anesthetized using 2% isoflurane and endo-tracheal intubation was carried out to ventilate the lungs while the chest was open. An incision of several mm length away from the sternal border towards the left armpit was made. Underlying connective tissue was opened and the two muscle layers underneath were spread and held by retractors without incision into the muscle. The rib cage was then visualized and opened at the 4^th^ intercostal space. The retractors were inserted and the pericardium was cut open to allow access to the heart. The left coronary artery was visualized running form the left atrium towards the apex as a red pulsating vessel [34]. The ligation was placed about 1 mm from the atrium to reach an infarction size of 40–50% of the ventricle. Next an 8–0 ligature was passed underneath the LAD and tied with three knots. For Sham operated animals, the pericardium was opened but no suture was inserted into the heart. The retractors were removed and the chest was closed by bringing together the 4^th^ and 5^th^ ribs using 6–0 nylon sutures. The muscles were placed into their original position and the skin incision was closed using a 6–0 nylon suture. Mice were monitored until awakening and body temperature was kept up by red light. After surgery mice were kept individually in double cages and sacrificed three or six days after surgery.

#### Laser Capture Microscopy (LCM)

Hearts were excised after perfusion with 40 ml Phosphate Buffer Saline (PBS) and immediately frozen in OCT at −80°C. PEN membrane coated slides (Carl Zeiss MicroImaging GmbH, Bernried, Germany) were activated and prepared as suggested by the supplier. Seven µm thick tissue sections of the left ventricle were obtained and washed for 10 seconds in DEPC-treated water, precooled 70% EtOH and 100% EtOH. Slides were air dried for 1–2 min. and stored at −80°C until processing. For laser dissection, slides were thawed briefly and excess liquid was removed. The epicardial samples, as well as cardiac muscle controls, were independently dissected out using a P.A.L.M. LCM (Carl Zeiss MicroImaging GmbH, Bernried, Germany) as shown in Figure S1.

For non-infarcted samples (sham operated), epicardial cells were obtained by dissecting the epicardial and subepicardial layer of the left ventricle. For infarcted samples, the infarction was carried out as described in the corresponding section and animals were sacrificed for dissection three or six days after induced infarction. Epicardial cells were dissected partly above and partly below the site of infarction using the infarct-suture site as landmark. Laser capture dissection represents the most reproducible way of collecting epicardial sample without introducing a bias towards certain cell populations (FACS sorting) or compromising RNA quality through prolonged tissue handling (staining). Fragments were catapulted into microtube lids filled with 65 µl RLT buffer (Qiagen, Milano, Italy) and 5 µl of carrier RNA (4 ng/µl solution).

### RNA isolation

After dissection, total RNA of the epicardial layer of individual hearts were isolated using a standard RNeasy micro kit (Qiagen, Milano, Italy) protocol. Quality of RNA was tested using Agilent Bioanalyzer 2100 (Agilent Technologies Inc., Santa Clara, USA). RIN values were at least above 6 while most samples had a RIN between 7 and 8. For each sample between 0.2 and 2 ng of RNA could be collected.

### Gene Arrays

RNA was processed for Affymetrix GeneChip analysis by the gene core facility at the European Molecular Biology Laboratory (EMBL) using a two cycle cDNA synthesis protocol as suggested by the supplier (Affymetrix, Santa Clara, CA) and a mouse430-2.0 chip (Affymetrix, Santa Clara, CA) was used for the analysis.

### Gene array data analysis and statistics

Data was processed and analysed using GeneSpring GX 7.3.1 (Agilent, Waldbronn, Germany). Gene array raw data was first normalized in order to make multiple chip data comparable using the pre-processing algorithm “GeneChip - Robust Multi-Array Average” (GC-RMA) method to account for background correction, normalization, and summarization. For further normalization values below 0.01 were set to 0.01. Each measurement was divided by the 50.0th percentile of all measurements in that sample. Each gene was divided by the median of its measurements in all samples. If the median of the raw values was below 10 then each measurement for that gene was divided by 10 if the numerator was above 10, otherwise the measurement was excluded. For each experimental group of tissues, samples from four or five animals were used. All array data is MIAME compliant and raw data has been deposited with ArrayExpress (Accession number: E-MEXP-2446).

### Cluster Analysis

Gene clusters representing similar expression profiles between uninjured epicardial and muscle control samples were identified using unsupervised, SOM clustering (Radius 7.0) with a maximum of 4500000 iterations. SOM clustering can be used to partition genes into clusters of similar gene expression changes across samples. In this analysis, only relative changes in expression levels are considered. Dimensions of 2×2 were chosen as this results in 4 clusters with discernible differences. Unsupervised SOM clustering for post infarction expression changes were carried out using an expanded 2×3 matrix, 10000 iterations, and a radius of 4.0. This expansion was necessary to account for the increased number of possible gene expression patterns that one can envision in the infarcted animals.

### Analysis of epicardial genes and differentially regulated genes post infarction

A list of WT epicardium-enriched genes was created by a parametric t-test using the Benjamini-Hochberg method to correct for multiple testing with a false discovery rate of 0.05 starting from SOM cluster 1 (from Figure 1). An epicardial signature gene list was derived from genes with a normalized >4-fold increased expression in the epicardial sample compared to the muscle control and a raw expression of at least 20 in the epicardial samples. We identified 197 unique genes that fulfilled these criteria and were thus considered epicardial signature genes. When comparing directly the mIGF-1 epicardial genes to WT signature genes, we applied statistical analysis for both as described above without prior use of unsupervised testing in order to maximize comparability between the two genotypes. For analysis of differentially regulated epicardial genes after infarction, clusters (3,1) and (1,2) were used. All statistical parameters and tests were used as described above.

### GO Analysis

For GO analysis, gene lists were analysed using the GOEAST online tool [35]. Hypergeometric testing was used with Benjamini & Yekutieli method for multiple testing correction using a maximum FDR of 0.005. For the analysis of the epicardial samples, the complete list of 197 epicardial signature genes was used as input (Table S1). For GO analysis of expression changes post infarction, the lists of statistically significantly regulated genes were used as input (Tables 3 and 4).

For further classification of the expression changes after infarction, KEGG analysis was also attempted, but no significantly enriched pathways could be identified.

### qRT-PCR

Isolated RNA was reverse transcribed using TaqMan Reverse transcription reagents (Applied Biosystems, Foster City, CA). Quantitative PCR was carried out using Applied Biosystems gene expression Taqman assays on a ABI PRISM 7700 Sequence Detector (Applied Biosystems, Foster City, CA). Samples were incubated at 50°C for 10 min, 95°C for 2 min and amplified for 40 cycles (95°C: 15 sec, 60°C: 1 min). Results were normalized against expression of the 18 S ribosomal RNA.

### Statistics and calculation for qRT-PCR

For quantitative reverse transcription PCR, expression was analysed by normalizing against expression levels of 18 S transcripts. To transform Ct values to relative expression values, the following formula was used :

Relative expression  = 2∧ -(ΔCt - Ct[stable]).

Where: ΔCt : Ct[Gene of Interest] – Ct[18 S]

Ct[stable]: Ct of one constant sample across all Ct's for the same gene.

Statistical significance was tested using student t-test (tail 2, type 2). Expression values are mean relative expression or mean fold induction ± standard error of the mean (SEM). A p value <0.05 was considered statistically significant unless noted otherwise. (*: p<0.05; **: p<0.005; ***: p<0.0005).

### Immunohistochemistry

Unfixed mouse heart sections were immersed at room temperature in 100% precooled Acetone for 10 minutes. Samples were washed three times with PBS for 3 minutes each and blocked with blocking solution containing 3% BSA in PBS for 30 minutes. Antibodies were applied at the appropriate dilution (rabbit anti-dermokine at 1∶250 (Abcam, Cambridge, MA), rat anti-GPM6A at 1∶100 (MBL International, Woburn, MA)) diluted in 3% BSA in PBS. Sections were incubated with primary antibodies overnight at 4 °C and washed three times in PBS for 5 minutes. Secondary antibody (Dermokine : Alexa goat anti rabbit 594 (Invitrogen, Carlsbad, CA); GPM6A - Alexa rabbit anti rat 594 (Invitrogen, Carlsbad, CA) was diluted in PBS at 1∶1000 dilution and incubated for 1 hr at room temperature. Slides were washed twice with DAPI (1∶20,000) in PBS for 5 minutes and mounted with Permafluor mounting medium (Beckman Coulter, Fullerton, CA). Basonuclin stains were carried out on paraffin embedded sections. Isolated tissues were fixed overnight at 4°C in 4% Formaldehyde/H2O solution. Tissues were gradually dehydrated (washes in PBS, 0.85% saline, 1∶1 saline/100%EtOH, 2×70% EtOH, 85% EtOH, 95% EtOH, 100% EtOH at 4°C). For further dehydration slides were incubated twice at room temperature in 100% Xylene and then 1∶1 xylene/paraffin, before they were subjected to 6 changes of 100% paraffin. Tissues were then placed into tissue block molds filled with paraffin. After letting stand over night to harden, 10 µm thick sections were cut using a microtome and placed on SuperFrost Plus microscope slides (Thermo Scientific, Germany). Slides were then left to dry at 42°C overnight. Before starting the staining protocol, slides were rehydrated through an ethanol series (100%>70%). They were then washed for one minute in dH2O. For antigen retrieval, the slides were placed in BG antigen retrieval solution (PickCell Laboratories, USA) and processed in a pressure cooker “Retriever 2100” (PickCell Laboratories, USA) as described in the protocol and left to cool down over night. Sections were incubated in 0.5% H2O2/dH2O for 10 minutes and then incubated in 5% normal horse serum for one hour at RT. After removing the serum, anti-basonuclin antibody (Santa Cruz) was applied at 1/50 dilution and incubated (1 h at RT for basonuclin antibody) in a moisturized chamber and washed three times in PBS. As secondary antibodies, peroxidase conjugated antibodies were used for 1 h at RT. Slides were then washed three times in PBS and were incubated in DAB substrate (SK4100, Vector Laboratories, USA) as described in the protocol. Slides were then rehydrated through an ethanol series (70%<100%) and mounted after three washes in Xylene.

## Supporting Information

Figure S1 Isolation of the epicardial cell layer of murine hearts using laser capture microscopy. A) Hearts were frozen and cut in a cryostat at 7µ¼m thickness, washed in DEPC treated water, 70% EtOH and finally 100% EtOH. B) The epicardial surface of the sections was laser excised (dissected area denoted E) and catapulted into RNA stabilizing solution for later RNA isolation. C) Cardiac muscle (excised area denoted M) was excised to compare gene expression of the epicardial layer to the rest of the heart. (Scale bar: 200µm).
(4.49 MB TIF)

Figure S2 Comparative analyses of epicardial signature genes. Epicardial data was compared to muscle control, left ventricle or whole heart gene array data. For each of the three control groups, a list of epicardial genes was generated using the same conditions. These lists were compared using a Venn diagram, showing that most genes were overlapping between the different comparison approaches (see Table S2). Especially highly expressed epicardial signature genes were found in all three approaches (white area), confirming our findings of epicardial genes.
(0.16 MB TIF)

Figure S3 Confirmation of post-infarction gene expression changes. RT-PCR analysis of epicardial signature gene expression changes post-infarct confirmed expression differences between three and six days post infarction were mostly negligible. In contrast, CD5L was upregulated three days post infarction but returned to basal expression levels after six days, highlighting the importance of immune response genes in early post infarction processes. GALDH was used as control. (S: Sham operated, 3: three days post infarction, 6: six days post infarction; *: p<0.05; ***: p<0.0005; errors are S.E.M.).
(0.36 MB TIF)

Table S1 Full List of epicardial signature genes.
(0.04 MB XLS)

Table S2 Comparative analyses of epicardial signature genes. Data supporting Figure S2.
(0.05 MB XLS)

Table S3 Full GO analysis of epicardial signature genes. Analysis of genes with a p-value cut off of 0.005.
(0.02 MB XLS)

Table S4 GO analysis of downregulated epicardial signature genes after myocardial infarction. Analysis of genes with a p-value cut off of 0.005.
(0.01 MB XLS)

Table S5 GO analysis of upregulated epicardial signature genes in muscle after myocardial infarction. Analysis of genes with a p-value cut off of 0.005.
(0.03 MB XLS)

Table S6 List of mIGF-1 epicardial signature genes.
(0.09 MB XLS)

## References

[1] Zhou B, Ma Q, Rajagopal S, Wu SM, Domian I (2008). Epicardial progenitors contribute to the cardiomyocyte lineage in the developing heart.. Nature.

[2] Cai CL, Martin JC, Sun Y, Cui L, Wang L (2008). A myocardial lineage derives from Tbx18 epicardial cells.. Nature.

[3] Reese DE, Mikawa T, Bader DM (2002). Development of the coronary vessel system.. Circ Res.

[4] van Wijk B, van den Berg G, Abu-Issa R, Barnett P, van der Velden S (2009). Epicardium and myocardium separate from a common precursor pool by crosstalk between bone morphogenetic protein- and fibroblast growth factor-signaling pathways.. Circ Res.

[5] Winter EM, Gittenberger-de Groot AC (2007). Epicardium-derived cells in cardiogenesis and cardiac regeneration.. Cell Mol Life Sci.

[6] Smart N, Risebro CA, Melville AA, Moses K, Schwartz RJ (2007). Thymosin beta-4 is essential for coronary vessel development and promotes neovascularization via adult epicardium.. Ann N Y Acad Sci.

[7] Christoffels VM, Grieskamp T, Norden J, Mommersteeg MT, Rudat C (2009). Tbx18 and the fate of epicardial progenitors.. Nature.

[8] Lepilina A, Coon AN, Kikuchi K, Holdway JE, Roberts RW (2006). A dynamic epicardial injury response supports progenitor cell activity during zebrafish heart regeneration.. Cell.

[9] Smart N, Risebro CA, Melville AA, Moses K, Schwartz RJ (2007). Thymosin beta4 induces adult epicardial progenitor mobilization and neovascularization.. Nature.

[10] Bock-Marquette I, Shrivastava S, Pipes GC, Thatcher JE, Blystone A (2009). Thymosin beta4 mediated PKC activation is essential to initiate the embryonic coronary developmental program and epicardial progenitor cell activation in adult mice in vivo.. J Mol Cell Cardiol.

[11] Riley PR, Smart N (2009). Thymosin beta4 induces epicardium-derived neovascularization in the adult heart.. Biochem Soc Trans.

[12] Limana F, Zacheo A, Mocini D, Mangoni A, Borsellino G (2007). Identification of myocardial and vascular precursor cells in human and mouse epicardium.. Circ Res.

[13] Winter EM, Grauss RW, Hogers B, van Tuyn J, van der Geest R (2007). Preservation of left ventricular function and attenuation of remodeling after transplantation of human epicardium-derived cells into the infarcted mouse heart.. Circulation.

[14] Winter EM, van Oorschot AA, Hogers B, van der Graaf LM, Doevendans PA (2009). A new direction for cardiac regeneration therapy: application of synergistically acting epicardium-derived cells and cardiomyocyte progenitor cells.. Circ Heart Fail.

[15] Hatcher CJ, Diman NY, Kim MS, Pennisi D, Song Y (2004). A role for Tbx5 in proepicardial cell migration during cardiogenesis.. Physiol Genomics.

[16] Kang J, Gu Y, Li P, Johnson BL, Sucov HM (2008). PDGF-A as an epicardial mitogen during heart development.. Dev Dyn.

[17] Lu J, Richardson JA, Olson EN (1998). Capsulin: a novel bHLH transcription factor expressed in epicardial progenitors and mesenchyme of visceral organs.. Mech Dev.

[18] Thorrez L, Van Deun K, Tranchevent LC, Van Lommel L, Engelen K (2008). Using ribosomal protein genes as reference: a tale of caution.. PLoS One.

[19] Moss JB, Xavier-Neto J, Shapiro MD, Nayeem SM, McCaffery P (1998). Dynamic patterns of retinoic acid synthesis and response in the developing mammalian heart.. Dev Biol.

[20] Niederreither K, Fraulob V, Garnier JM, Chambon P, Dolle P (2002). Differential expression of retinoic acid-synthesizing (RALDH) enzymes during fetal development and organ differentiation in the mouse.. Mech Dev.

[21] Xavier-Neto J, Shapiro MD, Houghton L, Rosenthal N (2000). Sequential programs of retinoic acid synthesis in the myocardial and epicardial layers of the developing avian heart.. Dev Biol.

[22] Lee JM, Dedhar S, Kalluri R, Thompson EW (2006). The epithelial-mesenchymal transition: new insights in signaling, development, and disease.. J Cell Biol.

[23] Zhang T, Li X, Yu W, Yan Z, Zou H (2009). Overexpression of thymosin beta-10 inhibits VEGF mRNA expression, autocrine VEGF protein production, and tube formation in hypoxia-induced monkey choroid-retinal endothelial cells.. Ophthalmic Res.

[24] Rosati B, Grau F, McKinnon D (2006). Regional variation in mRNA transcript abundance within the ventricular wall.. J Mol Cell Cardiol.

[25] Basset-Seguin N, Yancey KB (1992). [Cleavage fragments of C3 and of dermo-epidermal junction of normal human skin].. Ann Dermatol Venereol.

[26] Andoh A, Fujiyama Y, Hata K, Sumiyoshi K, Bamba T (1996). Regulation of complement C3 synthesis by interleukin-1 and transforming growth factor-beta in rat non-transformed intestinal epithelial cell line, IEC-6.. J Gastroenterol.

[27] Matsui T, Hayashi-Kisumi F, Kinoshita Y, Katahira S, Morita K (2004). Identification of novel keratinocyte-secreted peptides dermokine-alpha/-beta and a new stratified epithelium-secreted protein gene complex on human chromosome 19q13.1.. Genomics.

[28] Brass EP, Peters MA, Hinchcliff KW, He YD, Ulrich RG (2009). Temporal pattern of skeletal muscle gene expression following endurance exercise in Alaskan sled dogs.. J Appl Physiol.

[29] Zhang X, Tseng H (2007). Basonuclin-null mutation impairs homeostasis and wound repair in mouse corneal epithelium.. PLoS ONE.

[30] Vanhoutteghem A, Djian P (2004). Basonuclin 2: an extremely conserved homolog of the zinc finger protein basonuclin.. Proc Natl Acad Sci U S A.

[31] Alves CC, Carneiro F, Hoefler H, Becker KF (2009). Role of the epithelial-mesenchymal transition regulator Slug in primary human cancers.. Front Biosci.

[32] Santini MP, Tsao L, Monassier L, Theodoropoulos C, Carter J (2007). Enhancing Repair of the Mammalian Heart.. Circ Res.

[33] Limana F, Bertolami C, Mangoni A, Di Carlo A, Avitabile D Myocardial infarction induces embryonic reprogramming of epicardial c-kit(+) cells: role of the pericardial fluid.. J Mol Cell Cardiol.

[34] Tarnavski O, McMullen JR, Schinke M, Nie Q, Kong S (2004). Mouse cardiac surgery: comprehensive techniques for the generation of mouse models of human diseases and their application for genomic studies.. Physiol Genomics.

[35] Zheng Q, Wang XJ (2008). GOEAST: a web-based software toolkit for Gene Ontology enrichment analysis.. Nucleic Acids Res.

