# Supplementary figures and images for: Revealing New Mouse Epicardial Cell Markers through Transcriptomics
Source: PLoS One. 2010 Jun 28;5(6):e11429. doi: 10.1371/journal.pone.0011429 (PMC2893200; doi:10.1371/journal.pone.0011429)

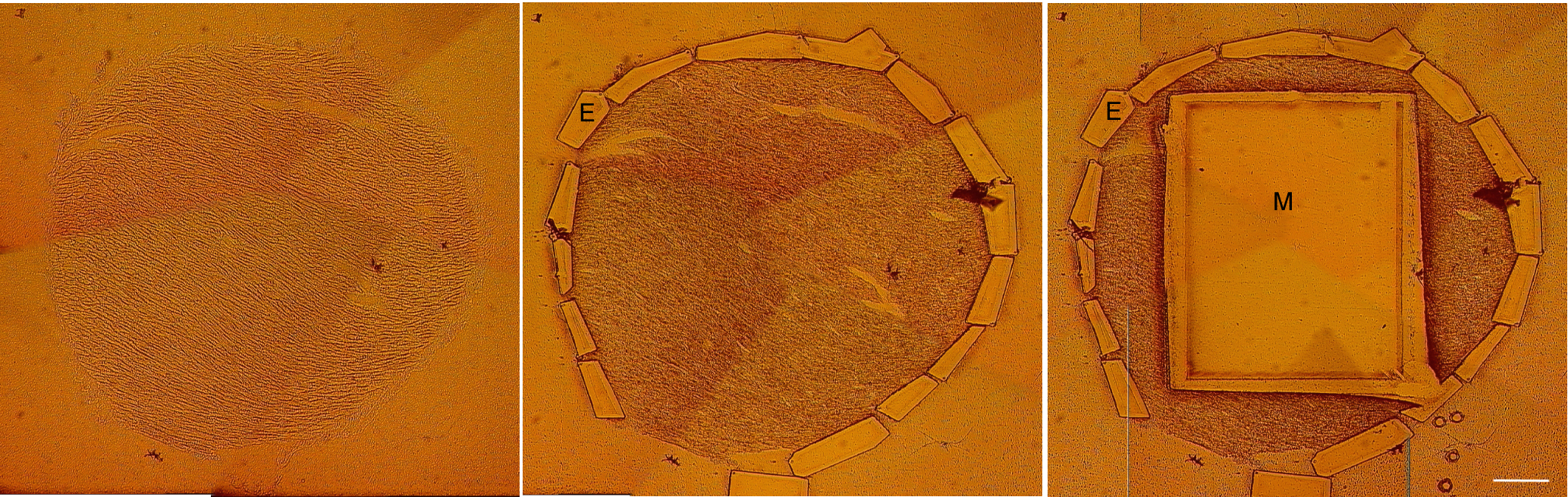

Supplement: Figure S1 — Isolation of the epicardial cell layer of murine hearts using laser capture microscopy. A) Hearts were frozen and cut in a cryostat at 7µ¼m thickness, washed in DEPC treated water, 70% EtOH and finally 100% EtOH. B) The epicardial surface of the sections was laser excised (dissected area denoted E) and catapulted into RNA stabilizing solution for later RNA isolation. C) Cardiac muscle (excised area denoted M) was excised to compare gene expression of the epicardial layer to the rest of the heart. (Scale bar: 200µm). (4.49 MB TIF) [file pone.0011429.s001.tif]

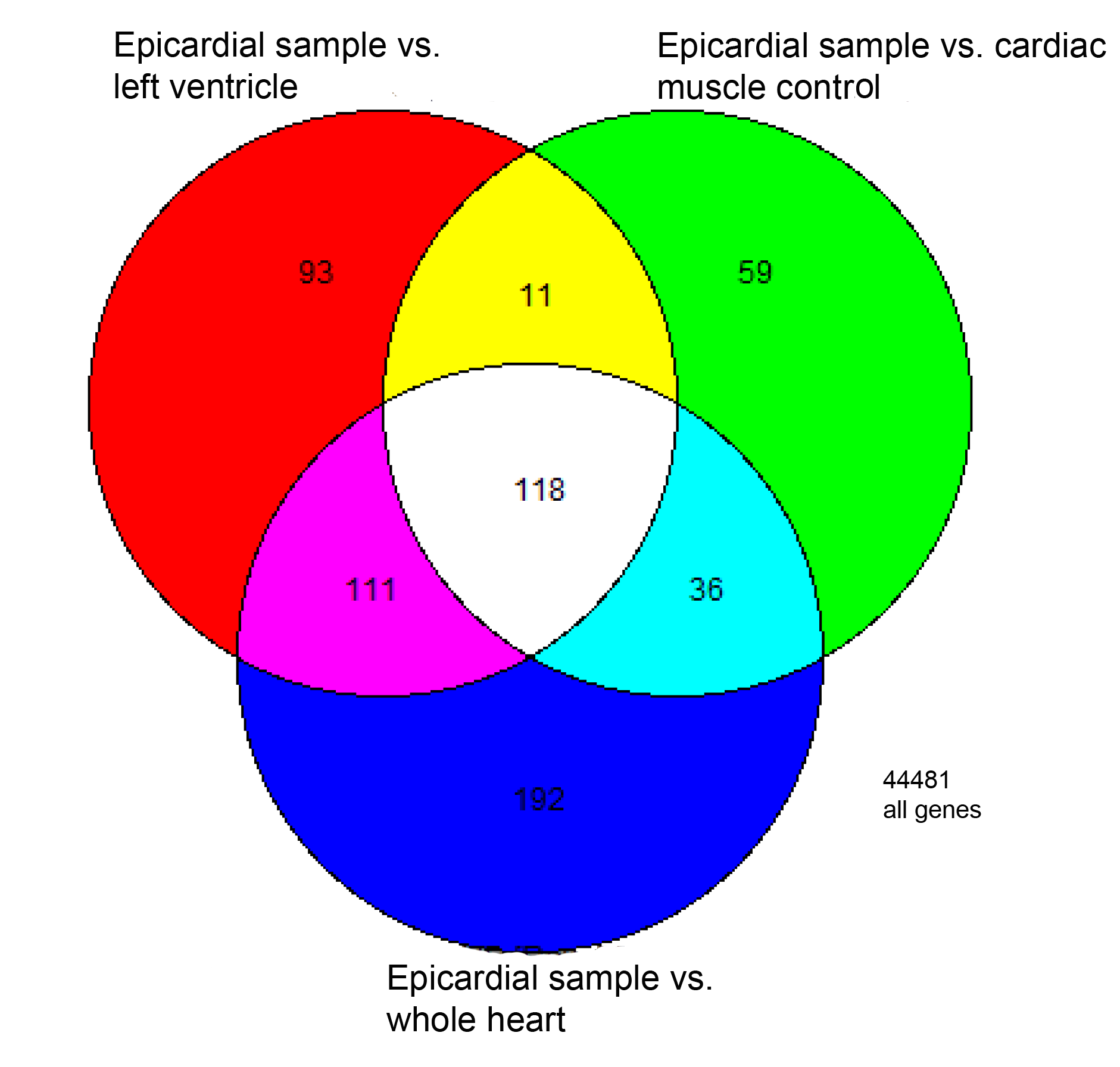

Supplement: Figure S2 — Comparative analyses of epicardial signature genes. Epicardial data was compared to muscle control, left ventricle or whole heart gene array data. For each of the three control groups, a list of epicardial genes was generated using the same conditions. These lists were compared using a Venn diagram, showing that most genes were overlapping between the different comparison approaches (see Table S2). Especially highly expressed epicardial signature genes were found in all three approaches (white area), confirming our findings of epicardial genes. (0.16 MB TIF) [file pone.0011429.s002.tif]

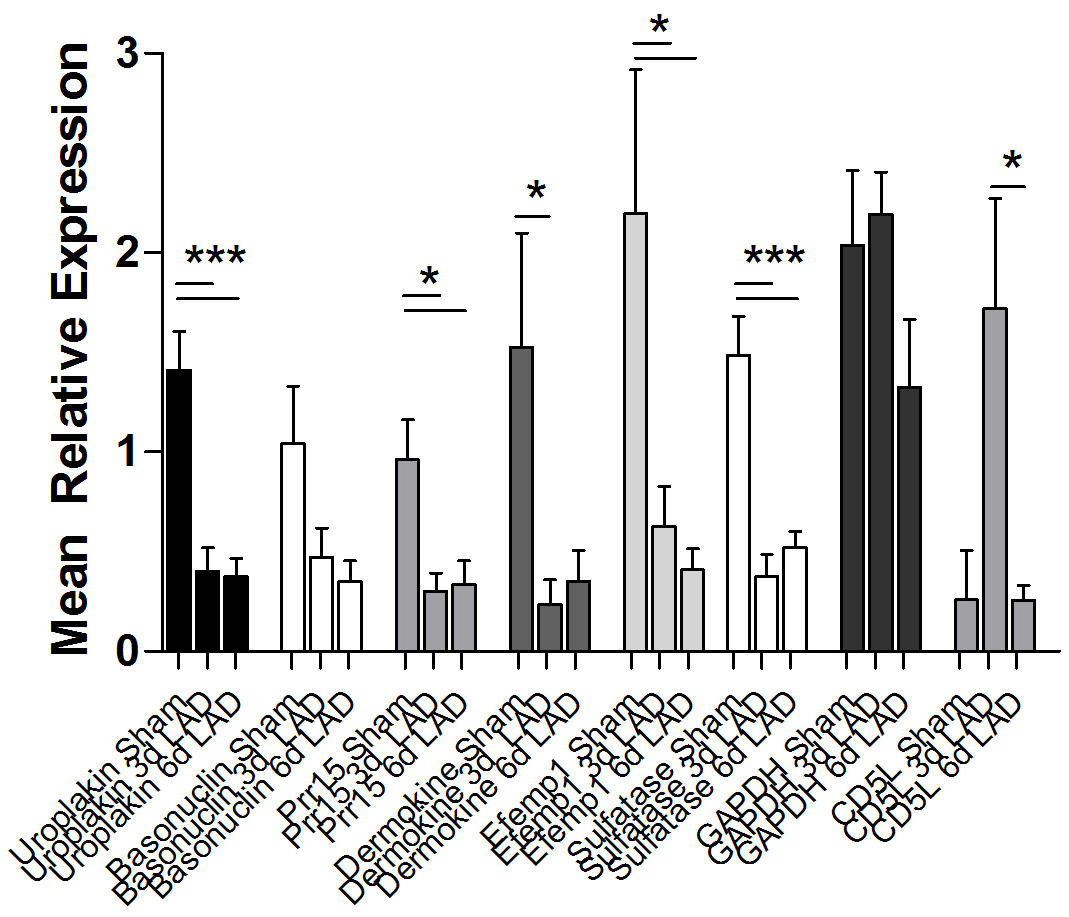

Supplement: Figure S3 — Confirmation of post-infarction gene expression changes. RT-PCR analysis of epicardial signature gene expression changes post-infarct confirmed expression differences between three and six days post infarction were mostly negligible. In contrast, CD5L was upregulated three days post infarction but returned to basal expression levels after six days, highlighting the importance of immune response genes in early post infarction processes. GALDH was used as control. (S: Sham operated, 3: three days post infarction, 6: six days post infarction; *: p<0.05; ***: p<0.0005; errors are S.E.M.). (0.36 MB TIF) [file pone.0011429.s003.tif]
